# Minimal Dose of Resistance Exercise Required to Induce Immediate Hypotension Effect in Older Adults with Hypertension: Randomized Cross-Over Controlled Trial

**DOI:** 10.3390/ijerph192114218

**Published:** 2022-10-31

**Authors:** Pedro Gargallo, José Casaña, Luis Suso-Martí, Ferran Cuenca-Martínez, Rubén López-Bueno, Lars Louis Andersen, Laura López-Bueno, Alba Cuerda-del Pino, Joaquín Calatayud

**Affiliations:** 1 Department of Physiotherapy, Faculty of Medicine and Health Science, Catholic University of Valencia, 46001 Valencia, Spain; 2 Exercise Intervention for Health Research Group (EXINH-RG), Department of Physiotherapy, University of Valencia, 46001 Valencia, Spain; 3 Department of Physical Medicine and Nursing, University of Zaragoza, 50009 Zaragoza, Spain; 4 National Research Centre for the Working Environment, 2100 Copenhagen, Denmark; 5 Department of Health Science and Technology, Aalborg University, 9220 Aalborg, Denmark; 6 Department of Physiotherapy, University of Valencia, 46001 Valencia, Spain

**Keywords:** hypertension, resistance training, post-exercise hypotension, volume

## Abstract

To determine the optimal exercise volume to generate a hypotension response after the execution of a single strength exercise in elderly subjects with hypertension (HT), a randomized crossover design was performed. A total of 19 elderly subjects with HT performed one control session and three experimental sessions of resistance training with different volumes in a randomized order: three, six, and nine sets of 20 repetitions maximum (RM) of a single elbow flexion exercise with elastic bands. The systolic blood pressure (SBP), diastolic blood pressure (DBP), and mean heart rate (MHR) were tested at the beginning and immediately afterwards, at 30 and 60 min, and at 4, 5, and 6 h after the resistance exercise. The results show that the volumes of six and nine sets of 20 RM obtained statistically significant differences in the SBP at 30 and 60 min post-exercise (*p* < 0.05); in the DBP at 30 min after exercise (*p* < 0.05); and in the MHR immediately after exercise at 30 and 60 min (*p* < 0.05), compared to a control session. A single resistance exercise with a minimum volume of six sets of 20 RM generated an acute post-exercise antihypertensive response that was maintained for 60 min in elderly people with controlled HT.

## 1. Introduction

Cardiovascular diseases are the leading cause of death in developed countries [1], with arterial hypertension (HT) being the main modifiable risk factor, responsible for 13% of all deaths in the world, with 45% of deaths from heart disease and 51% from stroke [2,3,4]. HT is prevalent in 40% of adults over 25 years [2], increasing the risk of having HT in people over 55 years by 90% [5]. Mainly due to lifestyle changes in the population, this unfortunate trend is expected to worsen in the years to come [6].

Regular exercise is the most effective non-pharmacological therapeutic approach to reducing blood pressure (BP) in older adults [7]. A recent meta-analysis showed that dynamic resistance exercise generates equal or greater decreases in resting BP than aerobic exercise in both normotensive and controlled or uncontrolled HT subjects [8]. In addition, this type of exercise is highly recommended among elderly patients due to its potential to minimize other consequences of aging, such as loss of strength, bone, and muscle mass and function [9].

The current literature suggests that the sustained antihypertensive effect generated by resistance exercise could be associated with the accumulation of successive episodes of acute decreases in BP that occurs after each session [10,11,12,13], a phenomenon known as post-exercise hypotension [14]. However, despite the clinical relevance of this phenomenon [10,12], the literature is scarce regarding whether this acute response occurs in older adults (>65 years) with HT controlled by medicine, especially after a single resistance training session.

To date, most resistance exercise studies in this field have included predominantly young [15], normotensive [16], and pre-hypertensive subjects [17]. In addition, scientific evidence shows great variability in the hypotensive response related to the resistance exercise heterogeneity in protocols, leaving a knowledge gap regarding the optimal or minimal dose to generate effective and long-lasting hypotensive effects. Parameters such as intensity, the number of muscles and amount of muscle mass involved, the type of muscle contraction, the order of exercises, rest intervals, the training method (circuit vs. traditional), the time of the day, the speed of execution, or the environment used (aquatic vs. land) have reported different post-exercise hypotension responses [10]. However, less is known regarding the role of exercise volume (understood as the product of the number of sets, repetitions, and exercises) on post-exercise hypotension. Only a few studies have compared different training volumes in older subjects [18,19,20,21], with most of the studies conducted in young adults [22,23,24,25]. These studies have indicated that a greater exercise volume leads to a stronger and longer antihypertensive response. However, most of these studies have analyzed the post-exercise hypotension of one vs. three sets in the very short-term (first 60 to 90 min), with no studies evaluating the immediate response to a single resistance exercise session, which could provide evidence on the minimum required dose to generate post-exercise hypotension, increasing applicability. Furthermore, all previous studies have used free weights or machines to train [19,20,21], something that could decrease adherence to the treatment among elderly patients [26,27].

The aim of the present study was to determine the minimal optimal amount of resistance exercise to generate post-exercise hypotension after the execution of a single exercise with the use of elastic bands in elderly patients with controlled HT. We hypothesized that a single resistance training exercise would have a hypotensive effect in older subjects with HT, and that a higher volume would result in a more effective and longer response than a lower volume.

## 2. Materials and Methods

### 2.1. Subjects

The study was carried out with subjects derived from a primary care center (Rocafort, Valencia, Spain). Demographic, anthropometric, and metabolic data were obtained from medical records.

The inclusion criteria were: HT (defined as systolic blood pressure (SBP) ≥ 130 mmHg or diastolic blood pressure (DBP) ≥ 80 mmHg, or taking antihypertensive drugs) [28] controlled with drugs, (at least during the last year), 55–70 years, sedentary lifestyle (less than 150 min of moderate physical activity per week and/or less than 75 min of vigorous physical activity a week), non-smokers, non-alcoholics, and signing the informed consent. Subjects had to maintain their normal type and dose of antihypertensive medication during the research period. Subjects were excluded in cases of serious renal, pulmonary, neurological, psychiatric, or cardiovascular disease (acute heart attack, stroke, peripheral artery disease, and ischemic heart disease), musculoskeletal limitations not allowing for exercise, or having participated in other exercise programs during the investigation or in the previous 3 months.

### 2.2. Procedures

This experimental study with a crossover design was conducted at a facility in the city where the primary care center was located between May (beginning of recruitment) and October of 2020. The research project was approved by the Ethics Committee of the University of Valencia (1045545), complying with the ethical requirements included in the Helsinki Declaration of 1975, revised in 2013. All participants signed the written informed consent. All sessions were carried out in the morning, at the same time, and evaluated by the same researcher, who was blinded to the training condition. The study was registered in ClinicalTrials.gov (NCT03957746) and adhered to the CONSORT guidelines to ensure transparent and standardized reporting of trials.

All subjects performed 5 sessions: 1 familiarization session to calculate the intensity, and 4 randomly-performed experimental sessions of resistance training with a biceps curl exercise (i.e., elbow flexion), with different volumes (1 condition per session and day). The simple randomization sequence was computer-generated (randomization.org) by an external member. Randomization was performed using a computer-generated random sequence table with a balanced block design (GraphPad Software, Inc., San Diego, CA, USA). An independent researcher generated the randomization list, and a member of the research team who was not involved in the assessment of the participants or the intervention was in charge of the randomization and maintained the list. The participants included were randomly assigned to one of the groups using the random sequence list, ensuring concealed allocation.

The 4 conditions of the study were: (1) 3 sets of 20 repetition maximum (RM); (2) 6 sets of 20 RM; (3) 9 sets of 20 RM; (4) control (rest for 18 min, that is, the time equivalent to performing 6 sets of 20 RM).

#### 2.2.1. Familiarization Session and Intensity Calculation

During the first visit, the subjects were familiarized with the evaluation and training procedures. In addition, a 20 RM test was performed to calculate the intensity for the different experimental sessions. To achieve adequate exercise intensity, the elastic bands were pre-stretched to approximately 50% of the initial length (initial length, 1.9 m), and then different bands were used/added when needed to reach the desired intensity. For this purpose, red, blue, black, silver, and gold elastic band colors were available (TheraBand CLX, The Hygenic Corporation, Akron, OH, USA), alone or combined in parallel. A maximum of 3–5 attempts were performed to achieve 20 RM.

#### 2.2.2. Experimental Sessions

The subjects were asked to avoid physical exercise and alcohol intake for 48 h before each experimental session. In addition, they were instructed to have a light breakfast 2 h prior to the session. The subjects did not receive recommendations about the diet to follow. The temperature of the facility where the investigation was carried out was kept between 21 °C and 24 °C during all the sessions [19].

The experimental sessions started 72 h after the familiarization session and were carried out 48 h apart, as in previous similar studies [19,20]. In all sessions, the same exercise was applied, at the same intensity, and with the same rest interval, differing only by the volume (number of sets). The exercise was performed unilaterally throughout the whole range of movement, alternating arms, with 1 min of rest between sets and with an execution rhythm of 1 s for the concentric part and 1 s for the eccentric part (without pause between phases), controlled with a metronome. The subjects were instructed to avoid the Valsalva maneuver during the exercise, and verbal feedback was provided when needed to maintain proper exercise technique [29]. The participants also received verbal encouragement when necessary for the last 3–5 repetitions to achieve 20 RM.

#### 2.2.3. Outcomes

The SBP (primary outcome) and the DBP and heart rate (HR) (secondary outcomes), were evaluated at the beginning of the session (after 10 min sitting at rest) and at different post-exercise time periods: immediately after finishing the exercise, at 30 min, and 60 min, as well as at 4, 5, and 6 h. The values of the variables evaluated at 4, 5, and 6 h were self-recorded at home after 10 min of sitting rest. The SBP and DPB measurements were taken in the left arm, following the recommendations of the American Heart Association [30], using a digital BP monitor (OMRON M7 Intelli IT, Kyoto, Japan). The subjects, in a sitting position, supported the left arm at the level of the heart, and the cuff to measure the BP was placed approximately 2.5 cm proximal to the ulnar fossa. Three measurements of the BP and HR were performed with 2 min of rest between them. The mean of the three measurements was the recorded value. The mean blood pressure (MBP) was calculated using the following formula: MBP = DBP + [SBP−DBP]/3 [22,31]. The PEH, having carried out a control session and experimental sessions, was calculated with the PEH_II method, defined as the difference between the BP after the intervention session and the BP after the control session. Thus, by including a control session as part of the experimental design and for the analysis of the PEH, any effect caused by the time variable (i.e., circadian effect) was reduced.

### 2.3. Statistical Analysis

Using repeated measures linear mixed models (Proc Mixed, SAS v.9.4; SAS Institute, Cary, NC, USA), differences between the conditions and time points were estimated. The condition, time, and condition by time were the predictors, controlling for the baseline value of the outcome variable. The results are presented as the least square means and the differences among the least square means. The effect size (Cohen’s d) was calculated and described as: <0.19 = trivial effect; 0.2–0.49 = small effect; 0.5–0.79 = moderate effect; >0.8 = large effect. Minimal clinically important differences were calculated according to a previous study by multiplying the pooled baseline standard deviation scores by 0.2 [32]. The sample size was estimated according to a previous study [33], where at least 14 patients were needed to obtain a post-exercise hypotension difference of 2 mm Hg for the SBP between exercise intensities and a residual SD of 2 mm Hg, with a statistical power of 0.80 and an alpha error of 0.05 (software G*power 3.1.0; University of Kiel, Kiel, Germany).

## 3. Results

### 3.1. Flow Diagram and Characteristics of the Sample

**Figure 1:**
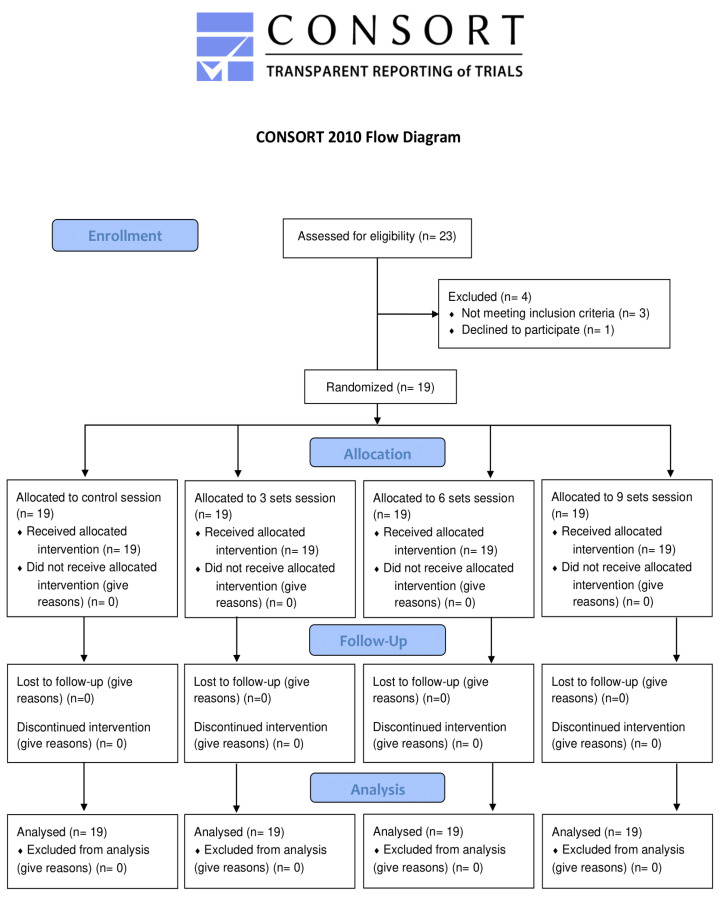
Flow diagram of the study.

The final sample was composed of 19 patients, including 13 men (68.42%) and 6 women (31.58%) (Figure 1). None of the patients had previous experience in resistance training, and all completed all the sessions without adverse events. Table 1 shows the anthropometric and metabolic characteristics of the subjects. There were no statistically significant differences among the baseline values of the SBP, DBP, HR, and BP in any of the experimental and control sessions. Most of the participants could be classified as grade I hypertensive subjects [28].

### 3.2. Hemodynamic Effects

Table 2 shows the mean values of the SBP and DBP in the different sessions at different measurement moments. The results obtained in the MBP and MHR are shown in Table 3. Table 4 shows the differences in the least square means (95% CI) of the SBP, DBP, and MBP of the control vs. the other conditions.

There were large and moderate significant hypotensive effects on the SBP with 6 and 9 sets, respectively, at 30 min, and moderate and large hypotensive effects 60 min after 6 and 9 sets, respectively. Regarding the DBP, trivial, moderate, and small significant hypotensive effects were observed immediately after finishing 3, 6, and 9 sets. In addition, the 6 and 9 sets extended the significant hypotension up to 30 min later, with a moderate magnitude. For the MBP, there was significant post-exercise hypotension after 6 and 9 sets at 30 and 60 min post-exercise, with small magnitudes. Significant reductions with small, trivial, and small magnitudes were also found in this variable immediately after 3, 6, and 9 sets, respectively. All the significant differences were clinically important. Table 5 shows the effect sizes and minimal clinically important differences from the interaction between the control and volume sessions. There were no HR differences among the different conditions at any time.

## 4. Discussion

### 4.1. Hemodynamic Effects

The main results obtained show that a single session composed of a single easy-to-perform resistance exercise for upper limbs with 6 or 9 sets and with very accessible equipment (elastic bands) produced post-exercise hypotension for 1 h in older adults with controlled HT.

The volumes of 6 and 9 sets produced a hypotensive effect on the SBP at 30 and 60 min post-exercise. However, at 30 min, the effect size was larger with 6 sets, while at 60 min, the effect size was larger with 9 sets. Moreover, a hypotensive effect was observed on the DBP immediately after finishing with 3, 6, and 9 sets, although to extend this response to 30 min post-exercise (with a moderate effect size), 6 and 9 sets were needed. In this way, the hypotheses initially raised are partially confirmed. Obtaining significant improvements in the DBP with a single exercise is therefore a positive fact to highlight.

Before carrying out the present study, there was little evidence of hypotensive effects after resistance exercises in older adults with HT since most studies have been conducted in healthy or normotensive subjects [15,16,17,34]. From the studies carried out with older adults, only four studies have compared the influence of volume on post-exercise hypotension in subjects with controlled HT [18,19,20,21]. In general, the results are in line with these previous studies, where higher volumes induced greater and longer post-exercise hypotension. For example, Brito et al. [19,20], when applying three sets of 10 repetitions of 10 exercises executed at 50% of 1 RM, obtained improvements in the SBP and DBP compared to one set up to the next 90 min of execution. This greater hypotensive response in comparison with the values obtained in the present investigation could be mainly due to the greater total volume of work and the greater baseline values recorded (~10–15 mm Hg higher). Likewise, Mediano et al. [18] found similar results at 60 min after exercise, but with hypotensive responses similar in terms of magnitude compared to the present study (possibly due to the lower number of exercises and baseline values) after comparing the effects of one vs. three sets of 10 repetitions of four strength exercises at maximum velocity. Finally, Scher et al. [21] also concluded that two sets generated higher post-exercise hypotension than one when performing a circuit resistance session (20 repetitions at 40% 1 RM, 10 exercises) during the first 60 min. However, in contrast with the present research, the authors found that the hypotensive response lasted beyond 60 min, probably due to the greater volume. In fact, if the total volume of repetitions is taken into account (number of sets x number of exercises x number of repetitions), the findings of the present work are especially relevant since post-exercise hypotension was obtained with volumes much lower than in previous studies (ranging between 100 and 400 total repetitions), which facilitates clinical application.

The total muscle mass involved was proven to be an important factor in post-exercise hypotension in young normotensive subjects [23]. However, the present study demonstrated that post-exercise hypotension can be induced with small muscle groups among the elderly population with controlled HT as long as the volume and intensity are appropriate. However, unlike the studies by Brito et al. [19,20] and Mediano et al. [18] who used between 4 and 10 resistance exercises, three sets with a single upper limb exercise were not enough to generate significant hypotensive responses in the SBP. It is necessary to emphasize that the findings obtained in the study also agree with studies on young subjects, where the greater the volume, the greater the magnitude of post-exercise hypotension [24].

Although determining the mechanisms associated with the hypotensive response generated by resistance training is beyond the scope of this study, it is possible that different isolated or combined physiological pathways have contributed to this phenomenon. Briefly, post-exercise hypotension associated with a strength workout seems to be related to a decrease in cardiac output and/or peripheral vascular resistance [35] (mainly the former in the case of older adults) [36], produced by a reduction in sympathetic activity [37], stroke volume [18], increased baroreflex sensitivity [38], and vasodilator substances, such as nitric acid and prostaglandins [39,40].

It is important to highlight the relevance of the results obtained in this study, as they show that a single resistance training session with different volumes can generate a significant acute hypotensive response in elderly subjects with controlled arterial HT. In addition, these hypotensive effects can be produced and maintained for 1 h after a single and extremely easy exercise of short sessions (18 min), with portable and cheap equipment that allows for home-based training. Therefore, the prescription of a resistance exercise with a minimum volume of 6 sets of 20 RM for older people with controlled HT would be sufficient if the main objective is to achieve a decrease in BP in the short term after training. Furthermore, the magnitude and duration of the response are of great importance, with reductions in the SBP and DBP higher than 2 mm Hg. Similar reductions may be sufficient to reduce cardiovascular risk, specifically the risk of death by stroke by 10% and by heart attack by 7% [4]. Furthermore, the results obtained can be considered clinically relevant since the magnitude of the reduction in the SBP and DBP with resistance exercise are similar to those obtained with antihypertensive medication (SBP: ~9.1 mm Hg; DBP: ~ 5.5 mm Hg) [41]. Likewise, the scientific literature indicates that the greater the decrease in post-exercise hypotension, the greater the chronic effects on BP [13], therefore making the protocol proposed in this investigation useful for older adults with controlled HT. Future studies should analyze these effects in a prolonged exercise program to determine the chronic implications of this kind of exercise on the hemodynamic parameters studied. Furthermore, while the sample size of this study was adequate for the intended objective, and it is in accordance with similar studies regarding PEH in older adults, this is a single-center project, so future multi-center investigations would be necessary to confirm and generalize the results obtained. In addition, analyzing the hypotensive response based on the type of antihypertensive medication could be an objective to consider in future research.

### 4.2. Study Limitations

Including a control session was a strength of the study. However, the evaluation of the possible mechanisms associated with post-exercise hypotension, such as HR variability, sympathetic activity, or nitric oxide production, could have provided interesting information. In addition, it seems logical that exercises involving greater muscle mass could have generated greater effects. However, the goal of the current study was to use an exercise as simple as possible, with the aim of maximizing its practical application in those without previous experience. Future studies should evaluate the influence of a greater number of sets and apply other changes in different training parameters to optimize prescription.

## 5. Conclusions

Performing six sets of 20 RM of a simple exercise with elastic resistance induced post-exercise hypotension lasting 60 min in older patients with controlled HT. This was the minimum volume necessary to maintain the response for 30 or 60 min, without obtaining a greater reduction when performing nine sets. However, nine sets conferred a greater effect size for systolic blood pressure reductions 60 min after exercise.

## Figures and Tables

**Table 1:** Anthropometric and metabolic characteristics of the sample.

Characteristics	Total Sample (n = 19)
Mean age (range) _1_	64.53 (55–70)
Gender (male) _2_	13 (68.42)
Height _1_	165.74 (7.37)
Weight _1_	80.37 (14.45)
BMI _1_	29.37 (5.37)
Diabetes _2_	9 (47.37)
Type I or II hypertension _2_	19 (100)
Antihypertensive medication _2_	19 (100)
Monotherapy	
Angiotensin-converting enzyme inhibitors (ACEI)	2 (10.52)
Angiotensin receptor blockers (ARBs)	1 (5.26)
Dihydropyridine calcium channel blockers (CCBs)	1 (5.26)
Diuretics	3 (15.78)
ẞ-Blockers	3 (15.78)
Angiotensin II receptor antagonist	1 (5.26)
Combination therapy	
Diuretics + CCBs	2 (10.52)
CCBs + ẞ-Blockers	1 (5.26)
ẞ-Blockers + ACEI	2 (10.52)
CCBs + ARBs	1 (5.26)
Diuretics + ẞ-Blockers+ ACEI	1 (5.26)
Diuretics + CCBs + ARBs	1 (5.26)

_1_ Mean (SD—standard deviation); _2_ absolute and relative frequencies (%); ACEI: angioten-sin-converting enzyme inhibitors; ARBs: angiotensin receptor blockers; BMI: body mass index; CCBs: dihydropyridine calcium channel blockers.

**Table 2:** Basal and post-exercise effects on SBP and DBP. Least square means (95% CI).

Time (min)	SBP (mm Hg)				DBP (mm Hg)			
	Control	3 Sets	6 Sets	9 Sets	Control	3 Sets	6 Sets	9 Sets
Basal	135 (130–140)	138 (133–143)	137 (132–142)	138 (133–142)	77 (75–80)	78 (76–81)	78 (76–81)	78 (75–81)
0	137 (132–142)	134 (129–139)	132 (127–137)	132 (127–137)	78 (75–81)	70 (68–73)	72 (69–75)	73 (71–76)
30	138 (133–143)	132 (127–137)	130 (125–135)	129 (124–134)	80 (77–82)	76 (73–78)	75 (72–78)	75 (73–78)
60	141 (136–146)	135 (130–140)	130 (125–135)	132 (127–137)	79 (76–82)	78 (75–80)	77 (74–79)	76 (73–78)
240	129 (124–134)	124 (119–129)	130 (125–135)	127 (122–132)	74 (71–76)	71 (69–74)	73 (70–76)	73 (70–76)
300	127 (122–132)	126 (121–132)	129 (124–134)	128 (123–133)	74 (71–76)	73 (70–76)	73 (70–75)	73 (70–76)
360	130 (125–135)	126 (120–131)	128 (123–133)	128 (123–133)	75 (72–77)	73 (70–76)	73 (70–76)	74 (71–77)

SPB: systolic blood pressure; DBP: diastolic blood pressure.

**Table 3:** Basal and post-exercise interventions on MBP and MHR. Least square means (95% CI).

Time (min)	MBP (mm Hg)				MHR (bpm)			
	Control	3 Sets	6 Sets	9 Sets	Control	3 Sets	6 Sets	9 Sets
Basal	97 (94–100)	98 (95–101)	98 (95–101)	98 (95–101)	68 (65–71)	68 (65–72)	68 (65–71)	68 (65–71)
0	98 (94–101)	92 (88–95)	92 (89–95)	93 (90–96)	65 (62–68)	70 (67–73)	72 (69–75)	73 (70–76)
30	99 (96–102)	95 (92–98)	93 (90–96)	93 (90–96)	63 (60–66)	65 (62–68)	65 (62–68)	65 (62–68)
60	100 (97–103)	97 (94–100)	95 (92–98)	95 (92–98)	61 (58–64)	62 (59–65)	63 (60–66)	63 (60–66)
240	92 (89–95)	89 (86–92)	92 (89–95)	91 (88–94)	69 (66–72)	70 (67–74)	72 (68–75)	67 (64–70)
300	92 (89–95)	91 (87–94)	91 (88–94)	91 (88–94)	69 (66–72)	73 (69–76)	73 (70–76)	68 (65–72)
360	93 (90–96)	90 (87–94)	91 (88–94)	92 (89–95)	71 (68–74)	72 (69–76)	71 (68–74)	67 (64–70)

CI: confidence interval; MBP: mean blood pressure; MHR: mean heart rate.

**Table 4:** Differences in least square means (95% CI) of SBP, DBP, and MBP of control vs. other conditions.

Time (min)	SBP			DBP			MBP		
	3 Sets vs. Control	6 Sets vs. Control	9 Sets vs. Control	3 Sets vs. Control	6 Sets vs. Control	9 Sets vs. Control	3 Sets vs. Control	6 Sets vs. Control	9 Sets vs. Control
0	03 (−04 to 10)	02 (−05 to 09)	02 (−05 to 09)	01 (−03 to 05)	01 (−03 to 05)	01 (−03 to 04)	02 (−03 to 06)	01 (−03 to 06)	01 (−03 to 05)
2	−03 (−10 to 04)	−04 (−11 to 03)	−05 (−12 to 02)	**−08 (−11 to −04)**	**−06 (−10 to −02)**	**−05 (−09 to −01)**	**−06 (−10 to −02)**	**−05 (−10 to −01)**	**−05 (−09 to −01)**
30	−06 (−13 to 01)	**−08 (−15 to −01)**	**−09 (−16 to −02)**	−04 (−08 to 00)	**−05 (−08 to −01)**	**−04 (−08 to −01)**	−05 (−09 to 00)	**−06 (−10 to −01)**	**−06 (−10 to −02)**
60	−06 (−13 to 01)	**−10 (−17 to −03)**	**−09 (−16 to −02)**	−01 (−05 to 02)	−02 (−06 to 01)	−03 (−07 to 00)	−03 (−07 to 02)	**−05 (−09 to −01)**	**−05 (−09 to −01)**
240	−05 (−12 to 03)	01 (−06 to 08)	−02 (−09 to 05)	−02 (−06 to 02)	−01 (−04 to 03)	00 (−04 to 03)	−03 (−07 to 02)	00 (−04 to 04)	−01 (−05 to 03)
300	−01 (−08 to 07)	02 (−06 to 09)	01 (−06 to 08)	−01 (−05 to 03)	−01 (−05 to 03)	−01 (−05 to 03)	−01 (−05 to 04)	00 (−05 to 04)	00 (−05 to 04)
360	−04 (−11 to 03)	−02 (−09 to 06)	−02 (−09 to 06)	−02 (−06 to 02)	−02 (−06 to 02)	−01 (−05 to 03)	−03 (−07 to 02)	−02 (−06 to 03)	−01 (−05 to 03)

Bold letters denote statistical significance. SBP: systolic blood pressure; DBP: diastolic blood pressure; MBP: mean blood pressure.

**Table 5:** Effect sizes of hemodynamic parameters after the different resistance volume sessions.

			Basal	0 min	30 min	60 min	240 min	300 min	360 min
SBP	Control vs. 3 sets	Magnitude	0.41	0.96	0.92	0.95	−0.26	−0.13	−0.28
		Classification	small	large	large	large	small	trivial	small
		MCID	3.75	3.25	3.05	3.62	7.26	6.17	8.39
	Control vs. 6 sets	Magnitude	0.35	0.82	0.93	0.79	0.27	0.81	0.02
		Classification	small	large	large	moderate	small	large	trivial
		MCID	3.31	3.10	2.73	3.24	5.15	3.14	4.06
	Control vs. 9 sets	Magnitude	0.41	0.80	0.79	0.90	0.12	0.74	6.65
		Classification	small	large	moderate	large	trivial	moderate	trivial
		MCID	3.24	3.31	2.65	3	3.14	2.58	6.51
DBP	Control vs. 3 sets	Magnitude	0.42	0.29	0.82	0.96	−0.36	−0.05	−0.23
		Classification	small	trivial	large	large	small	trivial	small
		MCID	2.06	2.11	2.07	2.18	4.38	3.80	4.94
	Control vs. 6 sets	Magnitude	0.30	0.63	0.67	0.60	0.49	0.67	0.00
		Classification	small	moderate	moderate	moderate	small	moderate	trivial
		MCID	2.11	1.93	1.81	2.03	3.08	1.81	3.75
	Control vs. 9 sets	Magnitude	0.23	0.45	0.74	0.68	0.53	0.47	0.09
		Classification	small	small	moderate	moderate	moderate	moderate	trivial
		MCID	1.70	1.87	1.73	1.97	2.11	1.62	3.82
MBP	Control vs. 3 sets	Magnitude	0.50	−0.21	−0.06	0.09	−0.48	−0.24	−0.25
		Classification	moderate	small	trivial	trivial	small	small	small
		MCID	2.16	2.04	2.03	2.22	5.25	4.43	5.97
	Control vs. 6 sets	Magnitude	0.37	-0.11	-0.31	−0.20	−0.16	0.28	0.00
		Classification	small	trivial	small	small	trivial	small	trivial
		MCID	2.19	1.97	1.84	2.09	3.57	1.91	4.57
	Control vs. 9 sets	Magnitude	0.39	−0.23	−0.38	−0.27	0.15	0.21	0.09
		Classification	small	small	small	small	trivial	small	trivial
		MCID	1.81	1.91	1.70	1.92	2.11	1.56	4.58
MHR	Control vs. 3 sets	Magnitude	0.23	0.44	0.04	−0.17	−0.31	0.01	−0.15
		Classification	small	small	trivial	trivial	small	trivial	trivial
		MCID	2.46	2.64	2.49	2.46	4.56	4.24	5.21
	Control vs. 6 sets	Magnitude	0.07	0.35	0.37	0.37	0.39	0.37	0.05
		Classification	trivial	small	small	small	small	small	trivial
		MCID	2.51	2.59	2.48	2.44	3.32	2.42	4.06
	Control vs. 9 sets	Magnitude	0.07	0.47	−0.05	−0.25	0.38	0.21	−0.07
		Classification	trivial	small	trivial	small	small	small	trivial
		MCID	2.53	2.92	2.57	2.57	2.23	2.33	3.82

DBP: diastolic blood pressure; MCID: minimal clinically important differences; SBP: systolic blood pressure; MBP: mean blood pressure; MHR: mean heart rate; MCID: minimal clinically important differences.

## Data Availability

Data supporting the results are available by sending an e-mail to the corresponding author.
